# The Anticariogenic Efficacy of Nano Silver Fluoride

**DOI:** 10.3389/fbioe.2022.931327

**Published:** 2022-07-01

**Authors:** C. Pushpalatha, K. V. Bharkhavy, Arshiya Shakir, Dominic Augustine, S. V. Sowmya, Hammam Ahmed Bahammam, Sarah Ahmed Bahammam, Nassreen Hassan Mohammad Albar, Bassam Zidane, Shankargouda Patil

**Affiliations:** ^1^ Department of Pedodontics and Preventive Dentistry, Faculty of Dental Sciences, M.S. Ramaiah University of Applied Sciences, Bangalore, India; ^2^ Department of Oral Pathology and Microbiology, Faculty of Dental Sciences, M.S. Ramaiah University of Applied Sciences, Bangalore, India; ^3^ Department of Pediatric Dentistry, College of Dentistry, King Abdulaziz University, Jeddah, Saudi Arabia; ^4^ Department of Pediatric Dentistry and Orthodontics, College of Dentistry, Taibah University, Medina, Saudi Arabia; ^5^ Restorative Department, Jazan University, Jazan, Saudi Arabia; ^6^ Restorative Dentistry Department, King Abdulaziz University, Jeddah, Saudi Arabia; ^7^ Department of Maxillofacial Surgery and Diagnostic Sciences, Division of Oral Pathology, College of Dentistry, Jazan University, Jazan, Saudi Arabia

**Keywords:** nano silver fluoride, silver diamine fluoride, anticaries agent, varnish, colloid

## Abstract

Dental caries is a common chronic disease, and anyone can be at threat of it throughout their lifespan. In school-aged children, dental caries is the most frequent disease related with oral health. Contemporary dental caries management focuses on non-restorative, non-invasive, and micro-invasive therapeutic techniques that effectively eliminate the caries progression at the lesion level and decrease the loss of healthy tooth structure. One of these strategies is to use caries apprehending agents with antibacterial and remineralizing characteristics. Due to recent regulatory approval in the United States, the use of silver diamine fluoride (SDF) for the therapy of dental caries has received substantial interest. SDF has successfully prevented and reversed both primary tooth caries and permanent teeth root caries. Even though SDF is an effective anti-caries agent, but it is associated with certain drawbacks like gum irritation, metallic taste, and irreversible dark stains on applying on cavities. As an alternative agent Nano Silver Fluoride (NSF) is preferable because it performs like SDF without tooth staining. It has comparable preventive and antibacterial activities as SDF. Further, it is ergonomic, economic and safe in children and adults. The current article aims to highlight the superior properties of NSF as a better anti-caries agent outstripping the limitations of discoloration of SDF.

## Introduction

Dental caries is a challenge since it is pervasive among children and has a detrimental influence on their quality of life. Dental caries is a worldwide public health concern that is consistently surveyed and reported in several countries. In 2020, the worldwide prevalence rate of dental caries in primary and permanent teeth was 46.2% and 53.8%, respectively, which was regarded as excessive (Kazeminia et al., 2020). A complicated interplay between acid-producing tooth-adherent bacteria and fermentable carbohydrates causes the dental caries. The acids in dental plaque may demineralize enamel and dentin in fissures and smooth surfaces of teeth over time. The so-called white spot lesion is the first visible symptom of dental caries. If demineralization continues, the white spot’s surfaces will cavitate, resulting in a cavity. White spot lesions may remineralize and not progress if the demineralization environment is decreased or removed. Therefore, effective measures should be undertaken to improve the condition described above, as well as to reconfigure and manage at all levels (Kazeminia et al., 2020). To achieve the objective of minimizing dental caries, it is necessary to seek for effective treatment and preventive treatment strategies. SDF has been used globally to combat dental caries in children, especially 38% solution. The presence of 38% SDF may decrease the demineralization of dentine and enamel and hinder the growth of cariogenic bacteria. Additionally, it prevents collagen breakdown in demineralized dentin (Mei et al., 2013a). SDF has a preventive impact on the complete dentition when just applied on carious anterior teeth and also proved successful in preventing cavities in permanent teeth (Chu et al., 2002; Mei et al., 2013a). Hence SDF therapy is considered as an indispensable component of caries prevention. According to published scientific clinical cases involving more than 4,000 young children globally, there is currently no indication of fatality or systemic detrimental consequences. SDF comprises approximately 24% and 28% (w/v) silver and 5%–6% (w/v) fluoride (Mei et al., 2013b). Oral exposure to one drop of SDF would result in a lower fluoride ion concentration than a 0.25 ml application of fluoride varnish (Crystal et al., 2017). Despite the benefits of SDF, there are disadvantages like as tooth discoloration, ulceration, and staining of tissues (Targino et al., 2014). These side effects instigated the researchers to find a material of equal efficacy while not compromising the esthetics. The application of nanoscience and technology in dentistry resulted in the emergence of Nano Silver Fluoride as a new anti-caries agent. Targino et al. (2014) first made NSF by chemically reducing silver nitrate, using chitosan as carrier and fluoride as stabilizing agent, while Haghgoo et al. (2014) made a varnish by mixing nanosilver with sodium fluoride varnish. Colloidal solution and varnish were the two most widely studied types of NSF. This novel anti-caries agent is safe for humans to use and has exceptionally significant antibacterial activity against Mutans streptococci and Lactobacilli, the primary microorganisms implicated in the formation of dental caries. The main composition of NSF proposed by Targino et al. (2014), was silver nanoparticles (376.5 μg/ml), chitosan (28,585 μg/ml), and sodium fluoride (5,028.3 μg/ml). The synergic components present in NSF formulation such as chitosan, AgNPs, and fluoride demonstrates that they are effective in caries prevention. The chitosan added into the formulation acts as stabilizing agent of the colloid with both antibacterial and anti-demineralizing effect. Fluoride incorporated into NSF also inhibits enamel mineral loss and has shown substantial capacity to suppress bacterial biofilm growth by its anti-adherence as well as anticariogenic activity. Silver is commonly used in the form of nitrates to achieve antibacterial effects. But when AgNPs are used, the surface area accessible for exposure to the microbial community is significantly increased. The antimicrobial effectiveness of nano silver particles is inversely related to their size. Sodium borohydride is frequently added as a reducing agent in preparing NSF formulations. In 2014, Haghgoo et al. (2014) prepared 5% NSF which was composed of Silver nanoparticle powder and polyvinyl pyridoline as a dispersant. It contained 22,600 ppm of slow release Sodium fluoride varnish stored in light proof brown bottle. Some researchers have used thiolated polyethylene glycol (PEG) as a reducing and capping agent in the formulation instead of sodium borohydride to lessen its toxicity. The PEG-coated AgNPs added into formulation enhances AgNPs stability even at high ionic concentrations. This modified formulation was shown to be less hazardous and less likely to oxidize (Zhao et al., 2020). NSF is available in the form of a yellow solution that has been shown to be stable over a three-year period. This material is both ecofriendly and affordable. The aim of this literature review is to present a brief narrative review of the existing literature on the anticariogenic efficacy of Nano Silver Fluoride.

## Anticariogenic Action of Nano Silver Fluoride

Anticariogenic action of NSF is associated with several processes, including reduced demineralization, accelerated remineralization, interference with pellicle and plaque development, and suppression of bacterial growth and metabolism. The cumulative effect of Chitosan, Silver nanoparticles, and Sodium fluoride added to the Nano Silver Fluoride formulation is responsible for the NSF anticariogenic action. The NSF colloidal formulation inhibits cariogenic biofilm formation, has antibacterial properties, and helps to remineralize teeth (Waikhom et al., 2022; Ahmed et al., 2019; Vieira Costa e Silva et al., 2018; dos Santos et al., 2014; dos Santos Junior et al., 2017). The anticariogenic effects of NSF have been surveyed through *in-vitro* and *in-vivo* studies against cariogenic microorganisms, as well as its remineralizing capability in both animal and human models which are discussed in the following headings.

### Anti-Bacterial Effect of Nano Silver Fluoride

**FIGURE 1:**
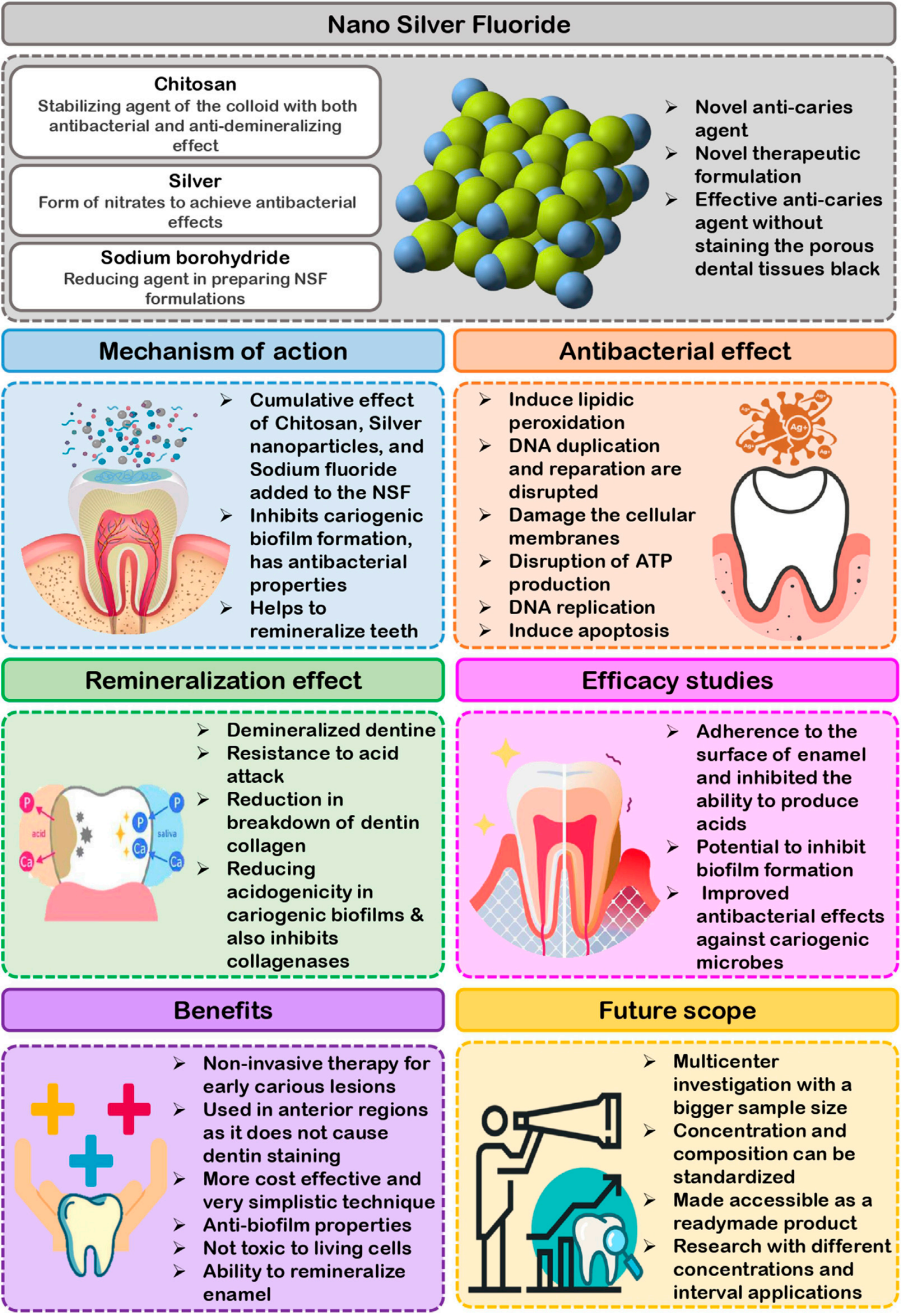
Highlights on Nano Silver Fluoride.

Studies reports that NSF is an excellent oral antibacterial agent because it is effective against cariogenic pathogens, primarily *Streptococcus mutans*, and also inhibits oral biofilm formation (Vieira Costa e Silva et al., 2018; Ahmed et al., 2019; Waikhom et al., 2022). Silver nanomaterials incorporated into NSF formulation are mainly necessary for the antibacterial property. The antibacterial activity of silver nanoparticles against *Streptococcus mutans* is 25 times stronger than chlorhexidine, particularly at diameters between 80 and 100 nm, while cytotoxicity has been found to rise at dimensions smaller than 20 nm (dos Santos Junior et al., 2017). Few studies have found AgNPs in NSF formulations with diameters of ranging from 2.56 ± 0.43 nm, 3.2 ± 1.2 nm and 5.9 ± 3.8 nm to favour antibacterial activity against *Streptococcus mutans* (Targino et al., 2014; dos Santos et al., 2014; Zhao et al., 2020). Studies reports that Silver nanoparticles (AgNPs) exhibits the antibacterial action by different mechanism. One such mechanism is by interrupting the bacterial cell wall and membrane integrity, encouraging the cell membrane permeability and loss of cell constituents, and ultimately inducing cell death (Shrivastava et al., 2007). AgNPs can inhibit the respiratory cascade by combining the sulfhydryl, causing lipid peroxidation, oxidative damage to DNA and proteins, and ultimately cell death (Hamed et al., 2017). AgNPs has a potential to attach to sulphur and phosphorous groups in DNA, resulting in DNA damage, aggregation, and disruption of transcription and translation (Durán et al., 2010). AgNPs promote dephosphorylation of phosphotyrosines, therefore interfering with cell signal transmission and thus damaging the cells (Shrivastava et al., 2007). When AgNPs are subjected to aerobic conditions, Ag+ may be released from the particles’ surface. The released Ag+ exerts powerful antimicrobial effects by direct interaction with the cell membrane and bacterial cell wall components, which is one of the most important mechanisms of AgNPs toxicity (Figure 1) (Xiu et al., 2012). Kim suggests that the production of free radicals by AgNPs could be assumed as one more way for AgNPs biocidal action (Kim et al., 2007). According to electron spin resonance spectroscopy study, when AgNPs come into contact with bacteria, they produce free radicals, which can damage the cellular membranes and make it porous, ultimately resulting in cell death (Sharma et al., 2018). According to Moronez’s research, the antibacterial activity of silver nanoparticles is size sensitive, with nanoparticles in the 1–10 nm range being more effective (Morones et al., 2005; Martínez-Castañon et al., 2008). Due to the fact that contact area and surface energy are inversely proportional to size, the smaller the silver nanoparticle, the greater its antibacterial activity. The antibacterial action of Chitosan, a potential agent used in the NSF formulation with polycation, is soluble in aqueous solutions of small organic acids like acetic acid and lactic acid and it can be linked in the mere existence of polyvalent anions like phosphates. This chitosan during cellular adherence process inhibits *Streptococcus mutans* and demonstrated significant antibacterial and plaque reduction activity at subsequent phases of accumulation, indicating that chitosan is effective in preventing dental caries (Hamed et al., 2017). Fluoride present in NSF formulation is effective at controlling cariogenic biofilms and reduces bacterial extracellular polysaccharide formation significantly. They are indeed effective at reducing acidogenicity in cariogenic biofilms and also inhibit collagenases, which slows the breakdown of dentin collagen (Gao et al., 2016).

### Remineralization of Enamel Carious Lesion by Nano Silver Fluoride

Fluoride added into the NSF formulation is mainly necessary for remineralizing early enamel carious lesion. Fluoride prevents caries largely through topical processes, such as preventing dental mineral dissolution by adsorbing onto crystal surfaces, encouraging remineralization at the crystal surfaces, and forming a fluorapatite coating that is acid resistant. The caries preventive effect of fluoride ions by its potency to establish the balance between demineralization and remineralization is crucial followed by the mitigation in the solubility of calcium hydroxyapatite (Oliveira et al., 2019). Silver nanoparticles can infiltrate into demineralized area and precipitate at that particular site resulting in an increase in the enamel hardness and resistance to acid attack (Rosenblatt et al., 2009). Chitosan, which is present in NSF formulations, has the potential to prevent tooth enamel demineralization by interfering with the release of enamel mineral elements. As a result, researchers suggested using nano silver fluoride containing silver NPs, Fluoride and chitosan is effective in enamel and dentin remineralization. Using optical coherence tomography and the microhardness test, researchers have found that nano silver fluoride seems to have a remineralizing effect on enamel caries. In addition, a clinical study revealed that nano silver fluoride is almost as efficient as silver diamine fluoride in preventing caries (Nozari et al., 2017; Teixeira et al., 2018; Silva et al., 2019). Another study also suggested that NSF could halt active dentine caries without causing tooth discoloration (Tirupathi et al., 2019). The silver and fluoride concentrations used in Dos Santos Jr.’s study were approximately 400 ppm and 2,275 ppm, respectively for remineralizing the demineralized dentine (dos Santos Junior et al., 2017).

## Therapeutic Use of Nano Silver Fluoride in Dental Caries Management

NSF is an anticaries agent that, when administered to enamel or dentin caries lesions, provides a non-invasive approach for caries arrest and treatment. Traditional dental therapy for the management of carious lesions may be time consuming and costly, and in some situations may not be practical owing to inability to afford or tolerate invasive treatment. NSF application is affordable, non-invasive and non-toxic and hence most communitis can afford it. The treatment approach is very simple; just two drops may be administered yearly to children without any risk. According to studies, NSF formulation is as effective as SDF in preventing and arresting dental caries (Nagireddy et al., 2019). NSF was compared to conventional antibacterial materials and SDF for its antibacterial efficiency against *Streptococcus mutans*, and it was found that NSF had lower Minimum Inhibitory Concentration (MIC) and Minimum Bactericidal Concentration (MBC) values than Chlorhexidine and SDF. As a result, lower NSF concentrations may be as effective as conventional products (Teixeira et al., 2018; Ahmed et al., 2019). Furthermore, NSF interfered with *Streptococcus mutans* adherence to the surface of enamel and inhibited the ability of *Streptococcus mutans* to produce acids more than Sodium Fluoride (Martínez-Castañon et al., 2008). Multidrug-resistant bacteria could be controlled by silver nanoparticles–fluoride colloids, which pose no significant hazard to human health. Nano-silver has a potential to inhibit biofilm formation by restricting biofilm-forming bacteria’s development. According to research, Nano-silver in varnishes has been found to have improved antibacterial effects against cariogenic microbes such *Streptococcus mutans* and *Streptococcus salivarius* (Di Giulio et al., 2013). NSF has been identified as an effective *Streptococcus mutans* biofilm inhibitor since it can lower the CFU counts (Teixeira et al., 2018; Vieira Costa e Silva et al., 2018; Espíndola-Castro et al., 2020a). As a result, the NSF formulation may be a more biocompatible antibacterial agent for *Streptococcus mutans*.

*In-vitro* studies have also proven that NSF has the greatest remineralization effect when compared against Nanohydroxyapatite serum and Fluoride varnish (Nozari et al., 2017). In a study analyzing the remineralization potential using Optical Coherence Tomography (OCT) assay found that NSF outperforms NaF and has established that silver nanoparticles added to NaF for enamel remineralization of primary dentition may augment fluoride performance because of their antibacterial effect (Vieira Costa e Silva et al., 2018). Further, NSF with 600 and 1,500 ppm were shown to create very little dentin staining than commercially available SDF in a digital spectrophotometric investigation comparing dentine staining induced by NSF and SDF (Espíndola-Castro et al., 2020a). Hence NSF may be a plausible substitute to SDF because it does not compromise esthetics. Moreover, NSF preparations maintain the collagen morphology and can induce mostly intrafibrillar mineral deposition with little extrafibrillar precipitation, characteristic of biomimetic remineralization and does not cause dentin discoloration while SDF caused a change in collagen morphology because of its high pH and dentin staining (Sayed et al., 2020).

According to Asmaa Aly Abo El Soud (2020), Nano silver fluoride (NSF) is more efficient than Silver Diamine Fluoride (SDF) on demineralized enamel surfaces of human premolars extracted for orthodontic reasons (Abo El Soud et al., 2020). NSF boosts remineralization by improving the intensity and quality of apatite crystal components. Nanosilver remineralized deciduous dental enamel and improved bactericidal activity without darkening the demineralized teeth with one exposure per year. In comparison to the usual sealant, AgNPs added to the sealant improved remineralization in permanent first molars’ and nano silver-based fluoride varnish preparation formulation were compared in an *in-vitro* study done by Targino et al. They found that antimicrobial efficacy of Nano silver based fluoride varnish formulation was superior to SDF. However, the cytotoxicity of Nano silver-based fluoride varnish preparation was lower than silver diamine fluoride (Targino et al., 2014). NSF at 10,147 ppm was not found to be as effective as sodium fluoride varnish (22,600 ppm) and SDF (44,800 ppm) on surface microhardness of enamel with artificial caries lesions (Akyildiz and Sönmez, 2019). The remineralization effectiveness of NSF in solution form was greater with increased surface microhardness values after enamel remineralization in comparison with NaF varnish and nano-Hydroxyapatite Serum group (Nozari et al., 2017). Zhao et al. (2020) suggested that NSF formulation containing 2.5% NaF and PEG-AgNPs had same remineralizing potential of dentinal caries in comparison to 12% SDF.

In underdeveloped countries, Nano Silver Fluoride (NSF) was a successful agent for preventing and reversing dental caries, but its influence on caries must be examined using alternate evaluation methods as well. The dental biofilm adhered to enamel treated with NSF had decreased numbers of *Streptococcus mutans* viability (absorbance) and colony forming units (CFU), and there was also a substantial distinction between the OHI-S mean values at baseline in a pilot randomised double blind crossover clinical study to evaluate the antimicrobial properties of NSF in 12 school children with their teeth treated with NSF in the experimental group and saline in the control group. Nanosilver fluoride was shown to be an efficient dental biofilm suppressor since it lowered *Streptococcus mutans* CFU counts and absorbance values, had no impact on biofilm pH, and decreased OHI-S values (Freire et al., 2017). Another study conducted in 130 decayed deciduous teeth of children treated with NSF and control (water), 81% of teeth in the NSF group had halted carious lesions on day 7, 72.7% after 5 months and 66.7% of lesions were still arrested after a year, whereas the control group had 0%, 27.4% and 34.7% respectively (dos Santos et al., 2014). In yet another study conducted in 22 children aged 1–6 years with white spot lesions detected by baseline DIAGNOdent values were treated with the fluoride plus 0.1% AgNPs and the children in control group were treated with commercial fluoride varnish once a week for a 3 weeks and after 3 months follow-up DIAGNOdent measurements were taken and it was found that teeth coated with AgNPs varnish exhibited lower mean fluorescence intensity than those treated generic varnish, indicating that dental remineralization was higher in this group (Butrón-Téllez Girón et al., 2017). Tirupathi et al. tested the therapeutic caries arresting potency of a prepared 5% silver nanoparticles introduced Sodium fluoride (NSF) varnish with commercially available thirty eight percent SDF in 159 primary molars in 50 children for a year and showed similar number of active and arrested caries. In primary molars, the authors discovered that a 5% NSSF therapy administered yearly is superior to or similar to a 38% SDF treatment in preventing dentinal caries (Tirupathi et al., 2019). In one of the double blinded randomized controlled trial done to compare the efficacy of different concentrations of NSF it was disclosed that higher concentrations of AgNPs in NSF attributed to the superior antibacterial efficacy. NSF when administered straight to dentinal caries, resulted in stoppage of cavities in 65.21% of teeth, and hence provides a minimally invasive approach for decay stoppage and cure (Nagireddy et al., 2019). Hence, from these in-vivo investigations, it can be concluded that NSF application can prevent tooth caries in around 65%–70% of instances, with no significant difference between NSF and SDF and has comparable clinical effectiveness as SDF in reducing the advancement of dentinal carious lesions in primary posterior teeth when applied once a year.In-vitro comparison of different fluoride-based varnishes such as SDF, NSF and propolis fluoride showed significantly increased level of calcium, phosphate, and fluoride ions on the treated human dentin discs surface. This suggests that NSF is a promising anticariogenic agent (Soekanto et al., 2017).

Table 1 highlights the *in-vitro* research and clinical trials exploring at NSF’s antibacterial impact, whereas Table 2 provides *in-vitro* and *in-vivo* remineralizing potential studies for caries management. Table 3 overviews *in-vivo* randomized controlled studies using NSF for caries management.

**TABLE 1:** Studies related to anti-microbial activity of NSF against cariogenic pathogens.

Author	Pathogens studied	Comparator	Tests done	Research findings
Teixeira et al. (2018)	*Streptococcus mutans*	NaF toothpaste	Microdilution tests, Anti-adherence and anti-acid effects	NSF had a MIC of 30 ppm against *Streptococcus mutans.* NSF-containing dentifrices out performed NaF-containing dentifrices in inhibiting bacterial attachment to the tooth surface (MIC of NaF dentifrice 180 ppm). NSF-containing dentifrices were able to avoid the pH drop
Vieira Costa e Silva et al. (2018)	*Streptococcus mutans*	NaF and Deionized water	Enamel adhesion and acid production	When compared to the negative control, NaF (positive control) and NSF were helpful at averting pH reduction, with NSF outperforming both the positive and negative controls
Haghgoo et al. (2014)	*Streptococcus mutans* and *Streptococcus salivarius*	Different concentrations of NSF (0.1%, 0.5%, 1%, 2.5%, and 5%)	Bacteria per millimeter	*Streptococcus mutans* and *Streptococcus salivarius* were both vulnerable to nanosilver varnish, with *Streptococcus salivarius* being more susceptible than *Streptococcus mutans*. In the presence of varnishes containing 1% nanosilver or higher, the population of microorganisms was reduced
Soekanto et al. (2017)	*Streptococcus mutans* and *Enterococcus faecalis*	Propolis fluoride and SDF	MIC, MBC	PPF had a 3% MIC for *Streptococcus mutans* and a 10% minimum bactericidal concentration (MBC). PPF had a MIC of 6% for *Enterobacter faecalis*. NSF’s MIC and MBC for *Streptococcus mutans* were 3.16% and 4.16%, respectively. For *Enterobacter faecalis*, the NSF MIC was 3.16%, whereas the MBC was 4.16%. Apparently NSF and PPF reduced biofilm development in a dose-dependent manner
Sayed et al. (2020)	*Streptococcus mutans* and *Streptococcus salivarius*	NaF varnish	Agar diffusion test	When the concentration of NSF was raised, the mean value of inhibition zone size (mm) rose. As the concentration of NSF was increased, the mean value of the inhibitory zone (mm) for *Streptococcus salivarius* decreased. In both bacterial strains, traditional fluoride varnish had no inhibitory zones
Gultom et al. (2019)	*Streptococcus mutans* and *Enterobacter faecalis*	SDF	Viability of Biofilm in various stages of maturation	NSF was comparable with Silver Diamine Fluoride in inhibiting formation of *Enterobacter faecalis* and *Streptococcus mutans* biofilm

**TABLE 2:** Studies related to remineralization potential of NSF for caries prevention.

Author, Year	Samples	Experimental group	Control group	Tests done	Research findings
Zhao et al. (2020)	Third molars	1%–12% SDF, 2%–2.5% NaF + AgNps	Deionized water	SEM, Micro CT	NSF and NaF proved equally successful in remineralizing enamel, with a statistically significant difference (*p* 0.001) when compared to deionized water, but no distinction is made between them
Teixeira et al. (2018)	Deciduous teeth	NSF NaF	Deionized water	Vickers microhardness, OCT	There is no statistically significant difference in VHN between the NSF and NaF dentifrices. OCT analysis demonstrates that the NaF and NSF dentifrices behave similarly
Nozari et al. (2017)	Primary anterior teeth	Group 1: 5% NaF varnish Group 2: n-HAP serum Group 3: NSF	Group 4: no agent	Vickers microhardness Atomic Force microscopy	NSF is perhaps the most effective in remineralization. In terms of remineralizing early caries, both NaF varnish and n-HAP serum performed similarly
Silva et al. (2019)	Human teeth	Group 1: NSF Group 2: NaF	No agent	Optic coherence Tomography, Vickers microhardness, Fluoresence spectroscopy	The extinction coefficients of the NaF and nanosilver fluoride groups were similar, but the negative group had a lower extinction coefficient
Ata (2019)	Premolars	Group 1: Control Group 2: NSF Group 3: N-HAP Group 4:CPP-ACP paste	No treatment	Vickers Microhardness	The highest SMH values were observed in NSF group (mean: 238.84 ± 20.31)
Nanda and Naik (2020)	Premolars	Group 3: NSF + GIC Group 4: NSF + composite	Group 1: GIC Group 2: Composite	Vickers microhardness	The mean microhardness value of GIC and composite groups pretreated with NSF was more than the non-treated group, indicating lesser demineralization
Akyildiz and Sönmez (2019)	Sound Third molars	NSF group Sodium Fluoride group SDF group	No treatment	Vickers Microhardness, SEM images	NSF group showed maximum mineral deposit on enamel surface
El-Desouky et al. (2021)	Primary teeth	NSF group (Ia) Fluoride Varnish Group (IIa)	No treatment (Ib, IIb)	Vickers Microhardness, Polarized Light microscopy	Both NSF and Fluoride varnish is equally effective

**TABLE 3:** *In-Vivo* randomized controlled studies related to NSF for caries management.

Author, Year	Study sample	Comparison	Research findings
Arnaud et al. (2021)	337 children aged between 5 and 7 years	NSF 600—Intervention and NSF 400—Positive control	When compared to NSF 400 (56.5%), NSF 600 had a greater performance level in preventing caries (72.7%, *p* = 0.025)
Freire et al. (2017)	12 school children of both genders, between 7 and 8 years	NSF- Experimental group Saline- Control group	In comparison to the other groups, NSF had lower CFU numbers. *Streptococcus mutans* growth in enamel treated with NSF is reduced
Nagireddy et al. (2019)	100 deciduous molars from 60 children	NSF- Experimental group Saline- Control group	After 7 days, 78% of teeth in the NSF group exhibited arrest of dentine caries, whereas 72.91% showed arrested caries after 5 months and 65.21% after 12 months
Tirupathi et al. (2019)	159 active dentinal carious lesions in primary molars (from 50 children)	5% Nano-silver fluoride varnish - Experimental group SDF—Control group	Success rate of 77% at the end of twelve-month follow up with Nano-silver fluoride varnish
Butrón-Téllez Girón et al. (2017)	22 children aged from 1 to 6 years	Experimental group- Fluoride varnish added with 0.1% AgNPs Control group- Commercial fluoride varnish	Teeth that had been lacquered with AgNPs showed a lower mean fluorescence intensity than those that had been painted with commercial varnish
dos Santos et al. (2014)	Primary teeth (130) in children	NSF—Experimental group Water- Control group	72.7% of the NSF group had halted decay, whereas 27.4% of the control group had 66.7% of lesions treated with NSF were still arrested after a year
Al-Nerabieah et al. (2020)	63 preschoolers with 164 active lesions	Combination of Nano-Silver Fluoride and Green Tea Extract (NSF-GTE) with SDF	At the end of 6 months, NSF-GTE and SDF had total arrest rates of 67.4% and 796

## Benefits of Nano Silver Fluoride

NSF can be used with minimal armamentarium even in peripheral community treatment camps. Since NSF is a non-invasive treatment for the management of early carious lesions, it will not create uncooperative behavior in very young children. It is safe to use in the anterior teeth without fear of tooth discoloration. NSF formulations do not oxidize when it comes in contact with the oxygen of the medium, hence is stable. There was no colour change noticed over time because of size of the AgNPs. Espíndola-Castro *et al* reported that yellowish stains are noticed after 2 weeks of NSF application mainly due the chitosan present in the formulation. However, these stains can be easily removed by gauze or by tooth brushing (Espíndola-Castro et al., 2020b). It does not irritate soft tissues since it has a lower pH and is more biocompatible. NSF is more cost effective than SDF and do not have metallic taste (Yin et al., 2020). Also, it has a very simple technique of application which can be learned by paramedical staffs at a Primary Health Center who can provide this treatment under a Dentist’s supervision. Nano silver particles have the ability to remineralize enamel, particularly in deciduous teeth even at a lower concentration. It is bactericidal to wide range of organisms like *Streptococcus mutans, Enterococcus faecalis,* and *Escherichia coli*. It has anti-biofilm properties as well. Nano silver fluoride is not toxic to living cells since majority of NSF formulations are prepared at a very low concentration (Akyildiz and Sönmez, 2019).

## Future Scope

To generalize the results, a multicenter investigation with a bigger sample size may be done. NSF concentration and composition can be standardized and made accessible as a readymade product. Primary health care personnel can be taught how to use NSF on children with high caries risk and from underserved populations. Research with different concentrations and frequencies of applications with larger sample size can be conducted to determine the best protocol for Nano-Silver Fluoride with natural extract application. Future *in vitro* and *in vivo* research can be conducted by integrating NSF into dental restorative materials to provide both antibacterial and remineralization advantages in a single material. This unique dental restorative material may prevent secondary caries, which is one of the most significant shortcomings in conventional restorative materials. Since NSF has excellent antibacterial property against wide range of pathogens it may be used in periodontal therapy to prevent and treat periodontal infections.

## Conclusion

NSF is an economic, ergonomic, non-invasive agent that is safer in children and adults. Also, it is biocompatible, non-discoloring, non-caustic and more efficient than the conventional fluorides and SDF. NSF can be used to treat decayed primary teeth in underdeveloped countries since it is simple, cheap, and does not require a sensitive application method. Thus, NSF is a better anticaries agent outstripping the limitations of SDF.

## References

[B1] Abo El Soud A. A. Elsaied H. A. Omar S. M. M. (2020). Comparative Evaluation of the Effects of Silver Diamine Fluoride (Commercial and Lab Prepared) versus Nano Silver Fluoride on Demineralized Human Enamel Surfaces (*In Vitro* Study). Egypt. Dent. J. (Oral Med. X-Ray, Oral Biol. Oral Pathology)) 66, 153–163. 10.21608/edj.2020.77529

[B2] Ahmed F. Prashanth S. Sindhu K. Nayak A. Chaturvedi S. (2019). Antimicrobial Efficacy of Nanosilver and Chitosan against Streptococcus Mutans, as an Ingredient of Toothpaste Formulation: An *In Vitro* Study. J. Indian Soc. Pedod. Prev. Dent. 37 (1), 46. 10.4103/jisppd.jisppd_239_18 30804307

[B3] Akyildiz M. Sönmez I. S. (2019). Comparison of Remineralising Potential of Nano Silver Fluoride, Silver Diamine Fluoride and Sodium Fluoride Varnish on Artificial Caries: an *In Vitro* Study. Oral Health Prev. Dent. 17 (5), 469–477. 10.3290/j.ohpd.a42739 31268047

[B4] Al-Nerabieah Z. Arrag E. A. Comisi J. C. Rajab A. (2020). Effectiveness of a Novel Nano-Silver Fluoride with Green Tea Extract Compared with Silver Diamine Fluoride: A Randomized, Controlled, Non-inferiority Trial. Ijdos 7, 753–761. 10.19070/2377-8075-20000148

[B5] Arnaud M. Junior P. C. Lima M. G. e Silva A. V. Araujo J. T. Gallembeck A. (2021). Nano-silver Fluoride at Higher Concentration for Caries Arrest in Primary Molars: A Randomized Controlled Trial. Int. J. Clin. Pediatr. Dent. 14 (2), 207–211. 10.5005/jp-journals-10005-1920 34413593 PMC8343678

[B6] Ata M. S. (2019). Influence of Nano-Silver Fluoride, Nano-Hydroxyapatite and Casein Phosphopeptide-Amorphous Calcium Phosphate on Microhardness of Bleached Enamel: *In-Vitro* Study. Tanta Dent. J. 16 (1), 25. 10.4103/tdj.tdj_29_18

[B7] Butrón-Téllez Girón C. Mariel-Cárdenas J. Pierdant-Pérez M. Hernández-Sierra J. F. Morales-Sánchez J. E. Ruiz F. (2017). Effectiveness of a Combined Silver Nanoparticles/fluoride Varnish in Dental Remineralization in Children: *In Vivo* Study. Superf. vacío 30 (2), 21–24. 10.47566/2017_syv30_1-020021

[B8] Chu C. H. Lo E. C. M. Lin H. C. (2002). Effectiveness of Silver Diamine Fluoride and Sodium Fluoride Varnish in Arresting Dentin Caries in Chinese Pre-school Children. J. Dent. Res. 81 (11), 767–770. 10.1177/154405910208101109 12407092

[B9] Crystal Y. O. Marghalani A. A. Ureles S. D. Wright J. T. Sulyanto R. Divaris K. (2017). Use of Silver Diamine Fluoride for Dental Caries Management in Children and Adolescents, Including Those with Special Health Care Needs. Pediatr. Dent. 39 (5), 135–145. 29070149

[B10] Di Giulio M. Di Bartolomeo S. Di Campli E. Sancilio S. Marsich E. Travan A. (2013). The Effect of a Silver Nanoparticle Polysaccharide System on Streptococcal and Saliva-Derived Biofilms. Ijms 14 (7), 13615–13625. 10.3390/ijms140713615 23812080 PMC3742206

[B11] dos Santos Junior V. E. Targino A. G. R. Flores M. A. P. Rodríguez-Díaz J. M. Teixeira J. A. Heimer M. V. (2017). Antimicrobial Activity of Silver Nanoparticle Colloids of Different Sizes and Shapes against Streptococcus Mutans. Res. Chem. Intermed. 43 (10), 5889–5899. 10.1007/s11164-017-2969-5

[B12] dos Santos V. E. Jr Filho A. V. Ribeiro Targino A. G. Pelagio Flores M. A. Galembeck A. Caldas A. F. Jr (2014). A New "Silver-Bullet" to Treat Caries in Children - Nano Silver Fluoride: A Randomised Clinical Trial. J. Dent. 42 (8), 945–951. 10.1016/j.jdent.2014.05.017 24930870

[B13] Durán N. Marcato P. D. Conti R. D. Alves O. L. Costa F. Brocchi M. (2010). Potential Use of Silver Nanoparticles on Pathogenic Bacteria, Their Toxicity and Possible Mechanisms of Action. J. Braz. Chem. Soc. 21 (6), 949–959. 10.1590/S0103-50532010000600002

[B14] El-Desouky D. Hanno A. Dowidar K. Hamza S. A. El-Desouky L. M. (2021). Evaluation of the Anticariogenic Effect of Nano Silver Fluoride on Demineralization of Enamel in Primary Teeth (An *In Vitro* Study). Alexandria Dent. J. 46 (2), 153–159. 10.21608/adjalexu.2020.20537.1017

[B15] Espíndola-Castro L. F. Rosenblatt A. Galembeck A. Monteiro G. (2020). Dentin Staining Caused by Nano-Silver Fluoride: a Comparative Study. Oper. Dent. 45 (4), 435–441. 10.2341/19-109-L 32053463

[B16] Espíndola-Castro L. F. Rosenblatt A. Galembeck A. Monteiro G. (2020). Dentin Staining Caused by Nano-Silver Fluoride: a Comparative Study. Oper. Dent. 45 (4), 435–441. 10.2341/19-109-L 32053463

[B17] Freire P. L. L. Albuquerque A. J. R. Sampaio F. C. Galembeck A. Flores M. A. P. Stamford T. C. M. (2017). AgNPs: The New Allies against S. Mutans Biofilm - A Pilot Clinical Trial and Microbiological Assay. Braz. Dent. J. 28, 417–422. 10.1590/0103-6440201600994 29160391

[B18] Gao S. S. Zhang S. Mei M. L. Lo E. C. Chu C. H. (2016). Caries Remineralisation and Arresting Effect in Children by Professionally Applied Fluoride Treatment - a Systematic Review. BMC oral health 16 (1), 12–19. 10.1186/s12903-016-0171-6 26831727 PMC4736084

[B19] Gultom F. P. Khoirunnisa N. Sahlan M. Soekanto S. A. (2019). Evaluation of the Potential of Nano Silver Fluoride against Streptococcus Mutans and Enterobacter Faecalis in Various Stages of Biofilm Maturation, AIP Conference Proceedings, Melville, USA (College Park, Maryland).

[B20] Haghgoo R. Saderi H. Eskandari M. Haghshenas H. Rezvani M. (2014). Evaluation of the Antimicrobial Effect of Conventional and Nanosilver-Containing Varnishes on Oral Streptococci. J. Dent. (Shiraz) 15 (2), 57–62. 24883341 PMC4033084

[B21] Hamed S. Emara M. Shawky R. M. El-domany R. A. Youssef T. (2017). Silver Nanoparticles: Antimicrobial Activity, Cytotoxicity, and Synergism with N-Acetyl Cysteine. J. Basic Microbiol. 57 (8), 659–668. 10.1002/jobm.201700087 28543603

[B22] Kazeminia M. Abdi A. Vaisi-Raygani A. Jalali R. Shohaimi S. Daneshkhah A. (2020). The Effect of Lavender (Lavandula Stoechas L.) on Reducing Labor Pain: A Systematic Review and Meta-Analysis. Evidence-Based Complementary Altern. Med. 2020 (1), 1–11. 10.1155/2020/4384350 PMC7673944 33224252

[B23] Kim J. S. Kuk E. Yu K. N. Kim J.-H. Park S. J. Lee H. J. (2007). Antimicrobial Effects of Silver Nanoparticles. Nanomedicine Nanotechnol. Biol. Med. 3, 95–101. 10.1016/j.nano.2006.12.001 17379174

[B24] Liu B. Y. Lo E. C. M. Chu C. H. Lin H. C. (2012). Randomized Trial on Fluorides and Sealants for Fissure Caries Prevention. J. Dent. Res. 91 (8), 753–758. 10.1177/0022034512452278 22736448

[B25] Martínez-Castañon G. A. Nino-Martinez N. Martinez-Gutierrez F. Martinez-Mendoza J. R. Ruiz F. (2008). Synthesis and Antibacterial Activity of Silver Nanoparticles with Different Sizes. J. nanoparticle Res. 10 (8), 1343–1348. 10.1007/s11051-008-9428-6

[B26] Mei M. L. Chu C. H. Lo E. C. M. Samaranayake L. P. (2013). Fluoride and Silver Concentrations of Silver Diammine Fluoride Solutions for Dental Use. Int. J. Paediatr. Dent. 23 (4), 279–285. 10.1111/ipd.12005 23033939

[B27] Mei M. L. Ito L. Cao Y. Li Q. L. Lo E. C. M. Chu C. H. (2013). Inhibitory Effect of Silver Diamine Fluoride on Dentine Demineralisation and Collagen Degradation. J. Dent. 41 (9), 809–817. 10.1016/j.jdent.2013.06.009 23810851

[B28] Morones J. R. Elechiguerra J. L. Camacho A. Holt K. Kouri J. B. Ramírez J. T. (2005). The Bactericidal Effect of Silver Nanoparticles. Nanotechnology 16, 2346–2353. 10.1088/0957-4484/16/10/059 20818017

[B29] Nagireddy V. R. Reddy D. Kondamadugu S. Puppala N. Mareddy A. Chris A. (2019). Nanosilver Fluoride-A Paradigm Shift for Arrest in Dental Caries in Primary Teeth of Schoolchildren: A Randomized Controlled Clinical Trial. Int. J. Clin. Pediatr. Dent. 12 (6), 484–490. 10.5005/jp-journals-10005-1703 32440060 PMC7229382

[B30] Nanda K. J. Naik S. (2020). An *In-Vitro* Comparative Evaluation of Pre-treatment with Nano-Silver Fluoride on Inhibiting Secondary Caries at Tooth Restoration Interface. Cureus 12 (5), e7934. 10.7759/cureus.7934 32494540 PMC7265756

[B31] Nozari A. Ajami S. Rafiei A. Niazi E. (2017). Impact of Nano Hydroxyapatite, Nano Silver Fluoride and Sodium Fluoride Varnish on Primary Teeth Enamel Remineralization: an *In Vitro* Study. J. Clin. Diagn Res. 11 (9), ZC97. 10.7860/JCDR/2017/30108.10694 PMC5713866 29207844

[B32] Oliveira B. H. Rajendra A. Veitz-Keenan A. Niederman R. (2019). The Effect of Silver Diamine Fluoride in Preventing Caries in the Primary Dentition: a Systematic Review and Meta-Analysis. Caries Res. 53 (1), 24–32. 10.1159/000488686 29874642 PMC6292783

[B33] Rosenblatt A. Stamford T. Niederman R. (2009). Oral Health Care in Disadvantaged Communities’ Oral Health Care in Disadvantaged Communities, 1999. J. Dent. Res. 88 (2), 116–125. 19278981 10.1177/0022034508329406

[B34] Sayed M. Hiraishi N. Matin K. Abdou A. Burrow M. F. Tagami J. (2020). Effect of Silver-Containing Agents on the Ultra-structural Morphology of Dentinal Collagen. Dent. Mater. 36 (7), 936–944. 10.1016/j.dental.2020.04.028 32475750

[B35] Sharma G. Nam J.-S. Sharma A. Lee S.-S. (2018). Antimicrobial Potential of Silver Nanoparticles Synthesized Using Medicinal Herb Coptidis Rhizome. Molecules 23 (9), 2268. 10.3390/molecules23092268 PMC6225489 30189672

[B36] Shrivastava S. Bera T. Roy A. Singh G. Ramachandrarao P. Dash D. (2007). Characterization of Enhanced Antibacterial Effects of Novel Silver Nanoparticles. Nanotechnology 18 (22), 225103. 10.1088/0957-4484/18/22/225103 37016550

[B37] Silva A. V. C. Teixeira J. D. A. Melo P. C. D. Lima M. G. D. S. Mota C. C. B. D. O. Lins E. C. C. C. (2019). Remineralizing Potential of Nano-Silver-Fluoride for Tooth Enamel: an Optical Coherence Tomography Analysis. Pesqui. Bras. em Odontopediatria Clínica Integr. 19. 10.4034/pboci.2019.191.50

[B38] Soekanto S. A. Rosithahakiki N. Sastradipura D. F. S. Sahlan M. (2017). Comparison of the Potency of Several Fluoride-Based Varnishes as an Anticariogenic on Calcium, Phosphate, and Fluoride Ion Levels. Int. J. Appl. Pharm. 9, 55–59. 10.22159/IJAP.2017.V9S2.14

[B39] Targino A. G. R. Flores M. A. P. dos Santos Junior V. E. de Godoy Bené Bezerra F. de Luna Freire H. Galembeck A. (2014). An Innovative Approach to Treating Dental Decay in Children. A New Anti-caries Agent. J. Mater Sci. Mater Med. 25 (8), 2041–2047. 10.1007/s10856-014-5221-5 24818873

[B40] Teixeira J. A. Santos Júnior V. E. D. MeloJúnior P. C. D. Arnaud M. Lima M. G. Flores M. A. P. (2018). Effects of a New Nano-Silver Fluoride-Containing Dentifrice on Demineralization of Enamel and streptococcus Mutans Adhesion and Acidogenicity. Int. J. Dent. 2018, 1351925. 10.1155/2018/1351925 29853891 PMC5964412

[B41] Tirupathi S. Svsg N. Rajasekhar S. Nuvvula S. (2019). Comparative Cariostatic Efficacy of a Novel Nano-Silver Fluoride Varnish with 38% Silver Diamine Fluoride Varnish a Double-Blind Randomized Clinical Trial. J. Clin. Exp. Dent. 11 (2), e105–e112. 10.4317/jced.54995 30805113 PMC6383905

[B42] Vieira Costa e Silva A. Teixeira J. A. Mota C. C. B. O. Clayton Cabral Correia Lins E. Correia de Melo Júnior P. de Souza Lima M. G. (2018). *In Vitro* morphological, Optical and Microbiological Evaluation of Nanosilver Fluoride in the Remineralization of Deciduous Teeth Enamel. Nanotechnol. Rev. 7 (6), 509–520. 10.1515/ntrev-2018-0083

[B43] Waikhom N. Agarwal N. Jabin Z. Anand A. (2022). Antimicrobial Effectiveness of Nano Silver Fluoride Varnish in Reducing Streptococcus Mutans in Saliva and Plaque Biofilm when Compared with Chlorhexidine and Sodium Fluoride Varnishes. J. Clin. Exp. Dent. 14 (4), e321–e328. 10.4317/jced.59093 35419182 PMC9000386

[B44] Xiu Z.-m. Zhang Q.-b. Puppala H. L. Colvin V. L. Alvarez P. J. J. (2012). Negligible Particle-specific Antibacterial Activity of Silver Nanoparticles. Nano Lett. 12 (8), 4271–4275. 10.1021/nl301934w 22765771

[B45] Yin I. X. Zhao I. S. Mei M. L. Lo E. C. M. Tang J. Li Q. (2020). Synthesis and Characterization of Fluoridated Silver Nanoparticles and Their Potential as a Non-staining Anti-caries Agent. Ijn 15, 3207–3215. 10.2147/ijn.s243202 32440119 PMC7212993

[B46] Zhao I. S. Yin I. X. Mei M. L. Lo E. C. M. Tang J. Li Q. (2020). Remineralising Dentine Caries Using Sodium Fluoride with Silver Nanoparticles: An *In Vitro* Study. Ijn 15, 2829–2839. 10.2147/ijn.s247550 32368057 PMC7185692

